# Examining the Impact of Case Management in Vancouver’s Downtown Community Court: A Quasi-Experimental Design

**DOI:** 10.1371/journal.pone.0090708

**Published:** 2014-03-05

**Authors:** Julian M. Somers, Akm Moniruzzaman, Stefanie N. Rezansoff, Michelle Patterson

**Affiliations:** Somers Research Group, Faculty of Health Sciences, Simon Fraser University, Burnaby, British Columbia, Canada; Erasmus University Rotterdam, Netherlands

## Abstract

**Background:**

Problem solving courts (PSC) have been implemented internationally, with a common objective to prevent reoffending by addressing criminogenic needs and strengthening social determinants of health. There has been no empirical research on the effectiveness of community courts, which are a form of PSC designed to harness community resources and inter-disciplinary expertise to reduce recidivism in a geographic catchment area.

**Method:**

We used the propensity score matching method to examine the effectiveness of Vancouver’s Downtown Community Court (DCC). We focused on the subset of DCC participants who were identified as having the highest criminogenic risk and were assigned to a case management team (CMT). A comparison group was derived using one-to-one matching on a large array variables including static and dynamic criminogenic factors, geography, and time. Reductions in offences (one year pre minus one year post) were compared between CMT and comparison groups.

**Results:**

Compared to other DCC offenders, those triaged to CMT (9.5% of the DCC population) had significantly higher levels of healthcare, social service use, and justice system involvement over the ten years prior to the index offence. Compared to matched offenders who received traditional court outcomes, those assigned to CMT (n = 249) exhibited significantly greater reductions in overall offending (p<0.001), primarily comprised of significant reductions in property offences (p<0.001).

**Conclusions:**

Our findings indicate that CMT achieved significantly greater reductions in recidivism than traditional court among offenders with complex needs and high numbers of previous offences. Limitations of this research include a non-experimental design and one year follow up. Strengths include a robust matching process and extensive client level data spanning multiple sectors. Further research is needed to replicate the observed outcomes, to investigate the extension of community courts to settings with divergent offender needs and local resources, and to estimate potential cost avoidance attributable to this intervention.

## Introduction

Problem solving courts have expanded greatly in number over the past thirty years [1]. Through a variety of collaborative practices, these courts diverge from the traditional administration of justice and aim to reduce criminal recidivism by addressing the factors that place individuals at risk for offending [2]. The various titles and foci of problem solving courts are shaped by the characteristics of the offender populations that they serve. These populations are sometimes identified on the basis of specific health conditions (e.g., mental health court, drug treatment court), social context (e.g., family court), or developmental status (e.g., youth court). The populations served by community courts are defined by geography and offence type – that is, they receive individuals charged with certain offences in a particular catchment area.

Following the implementation of the first community court in Midtown Manhattan in 1993, similar courts have proliferated in the United States, South Africa, England, Wales, and Australia [3]. Canada’s first community court was implemented in Vancouver in 2008. The practices and staffing of different community courts vary in response to the needs in the local offender population [4], which can include individuals who are homeless, mentally ill, substance dependent or military veterans. In general, community courts aim to act swiftly, and to create opportunities for restitution and community service [5].

### Evaluations of Community Courts

Among the evaluations generated to date, authors have reported that community courts are associated with greater use of alternative sanctions [6] and that offenders report higher perceptions of “fairness” compared to traditional adjudication [7]. Although encouraging, these studies do not address the fundamental question of whether community courts are effective at reducing reoffending, and thereby at improving community safety. Very little of the literature concerning community courts has been published, and no studies of recidivism have yet appeared in peer reviewed journals. A review of the available research on community courts described the literature as “shockingly sparse” [8] (p.261). The need for empirical research is amplified by the prospect that community courts may expand in a manner similar to the growth of other problem-solving courts [9].

Given the heterogeneity across communities, it is not surprising that community courts have developed a wider range of practices than courts that serve a relatively standardized offender group, such as drug treatment courts. However, this variability only increases the need for outcome research, in order to create knowledge about the diversity of settings where community courts may be effective, and the adaptations and enhancements that may be required in order to support change with particular groups of offenders or in particular contexts such as suburban or rural locations.

A challenge confronting research on problem solving courts in general is the need for valid comparison groups. Offenders who enter problem solving courts may be distinguished from the general offender population on the basis of several important characteristics. In addition to meeting the specific criteria for entry into the court (e.g., geography, substance use, mental illness), offenders must often agree to plead guilty or otherwise choose to pursue an alternative to traditional adjudication. Community courts have been implemented in settings where complex social problems (e.g., poverty, homelessness, unmet health needs, etc.) are intertwined with high levels of street crime. For these reasons, it is not valid to compare outcomes for offenders in community court (or other problem solving courts) with outcomes among offenders in more traditional courts. The design that best addresses the challenge of creating a valid comparison group is random assignment. However, this approach is relatively complex to implement, and may not be legally viable because it entails eliminating the choices of clients and their lawyers concerning the type of court that they would be assigned to. In addition, randomization does not address the role of client motivation, and the fact that choosing to enter a problem solving court may signify that a client is ready to undertake changes in their life.

Quasi experimental designs have been used to examine outcomes in a number of problem solving courts, including drug treatment courts [10] and mental health courts [9]. These studies use the technique of propensity score matching (PSM) to minimize the impact of confounding variables in observational research [11]. PSM involves matching individuals concurrently on a number of characteristics, and the effectiveness of the procedure increases with the inclusion of multiple variables that are each related to the outcome of interest.

A variety of measures have been used to represent “recidivism” in studies of problem solving courts, including number of jail days post-discharge [12], number of arrests, rearrests and/or offence severity [13]–[15], and prosecutorial action [16]. Following completion of a community court program there may be some interactions with the justice system (e.g. police contact) that are not necessarily evidence of negative outcomes. However, a reduction in criminal convictions may be interpreted as relatively clear evidence of improved outcomes for individuals and for communities.

### Vancouver’s Downtown Community Court

Vancouver’s “Downtown Community Court” (DCC) was created on the recommendation of the provincial Justice Review Task Force [17]. The court was identified as a key strategic priority in response to longstanding problems related to crime in Vancouver’s downtown. As in other settings where community courts have been introduced, the DCC recognizes that many (though not all) offenders in the community face challenges that may increase their risk of offending, and considers that the neighbourhood itself includes resources that can be integrated to assist offenders in ways that may reduce the risk of recidivism while enhancing the overall health and safety of the community [18]. Extensive planning preceded the implementation of the court, influencing its design and operations. Over fourteen agencies collaborate in the DCC, representing health, justice, and social services.

The DCC hears all but the most serious offences committed in the downtown catchment area, the most common type being theft [17]. Offenders are not required to plead guilty in order to enter the DCC. The court provides sanctions, services and interventions to reduce the risk of reconviction. The resources available to the DCC include a triage team and representatives of health, justice, and social welfare agencies who are dedicated to work with DCC participants. The majority of offenders in the DCC have their cases resolved with sanctions (that frequently include community service) and are supervised in the community by a probation officer [17]. Offenders with more complex needs and higher criminogenic risk are assigned to a Case Management Team (CMT). The CMT consists of four probation officers, two staff members from the local health authority, two staff from the ministry responsible for social assistance, and one police officer. Team members are collectively able to respond to diverse and often overlapping needs among offenders, including housing, healthcare, addiction treatment, income assistance, and vocational assistance. Additional resources assigned to the CMT are: one Aboriginal court worker, one housing worker, one victim support worker, and one forensic psychiatrist. The case management team oversees the completion of community service requirements as well educational and counseling sessions. The CMT assertively supports offenders who require a high level of assistance managing one or more significant changes related to their offence risk, such as rehousing, gaining employment, accessing healthcare, or changing peer group involvements.

### Approach and Hypotheses

The present study investigates the impact of the CMT on recidivism among participants. We focus on the subset of DCC offenders who were assigned to the CMT because of the expected high level of criminogenic risk and need within this group. Our design does not address outcomes for the larger number of DCC cases that are not triaged to CMT. Random assignment to conditions was not possible. We derive a comparison group using the PSM technique, using a large array of variables that represent both static and dynamic risk factors associated with recidivism. We incorporate an intent-to-treat (ITT) method, including all participants who were triaged to CMT, with 12 months of follow up after exiting the CMT program. We hypothesize that reductions in recidivism (one year pre minus one year post) will be significantly greater in the CMT group than in the matched comparison group.

## Methods

### Ethics Statement

This study was approved by the Research Ethics Board of Simon Fraser University.

### Data Sources

This study used non-identifying data provided through the British Columbia Inter-Ministry Research Initiative (IMRI). The purpose of the IMRI is to produce knowledge that supports the development and evaluation of multi-agency programs involving the justice sector. We examined linked administrative data, spanning three provincial government ministries: Justice; Health Services; and Social Development & Social Innovation.

Data from the contributing ministries comprise a relatively complete inventory of the health, justice, and income assistance services used by members of the British Columbia population. The completeness of these data reflects the central organizational and funding role provided by the provincial government in the administration of these various services. The IMRI is governed by Information Sharing Agreements between the partnering ministries and the host university. Planned analyses are reviewed and developed by a Steering Committee with representatives from each of the partnering institutions. Access to data is subject to police security clearance, restricted to a designated secure off-line environment and other provisions to protect privacy. The current analysis uses linked data spanning from 1997 to 2013.

### Participants

Our analysis included all individuals enrolled in the DCC since the court’s inception in 2008 up to 2011. We then selected those individuals who were triaged to the CMT, regardless of the duration of their involvement with the program.

A propensity score was calculated for the DCC cohort using the following variables.

1. Demographics: age, gender, ethnicity, education.
2. Correctional history in the ten years prior to DCC: number of total offences; number of property offences; number of violent offences; number of offences involving weapons; number of breach offences; number of sentences involving custody.
3. Community health services in the five years prior to DCC: costs of community medical services overall; number of community medical service encounters for substance-related mental disorders; number of community medical service encounters for non substance-related mental disorders; number of community medical services.
4. Hospital services in the five years prior to DCC: number of admissions; days in hospital.
5. Social assistance in the five years prior to DCC: total number of payments; amount paid for disability, hardship, or any other benefit.

Matching variables were chosen to reflect both static and dynamic factors associated with the risk of offending.

### Baseline and Follow-up Periods

In order to calculate baseline values for offences, we included all convictions occurring in the one-year period prior to enrolment in the DCC for the intervention group, or the one-year period prior to sentencing for the comparison group. For DCC participants, follow-up began when they exited the CMT. DCC participants who exited the program after March 31, 2012 were excluded to ensure at least one year of follow up. For the comparison group, the follow up period consisted of the one year following index sentencing.

### Comparison Group Participants

Eligibility for inclusion in the comparison group was restricted to individuals who had been sentenced through the Vancouver Provincial Court that is located adjacent to the DCC in the Downtown Eastside of Vancouver. The Provincial Court receives individuals from the urban area surrounding the geographic catchment of the DCC. Individuals were eligible for inclusion if they were sentenced between April 1 2008 and March 31 2011. The Vancouver Provincial Court saw individuals charged with offences in Vancouver surrounding the catchment area for the DCC. These restrictions were instituted to ensure a contemporaneous sample from the same location, thereby having comparable access to publicly available community services and supports.

### Statistical Analysis

The Propensity Score Method (PSM) was applied to identify the comparison group from the comparison pool using the nearest neighbor technique (one-to-one matching), without replacement with a caliper of 0.05. Propensity score (the predicted probability) was obtained from multi-variable logistic regression using CMT membership as a dependent variable and all the matching variables as predictors. Matching variables for multi-variable logistic regression were chosen based on statistical significance in bi-variate analysis as well as evidence in existing literature. Due to improved model fitness statistics, service use variables were chosen for the last five years while correction-related variables were chosen for the ten years preceding the index offence.

The STATA module ‘PSmatch2’ was used to draw the comparison group and to check balances of matching variables [19]. Parametric and non-parametric tests (Independent sample t test and Pearson Chi-square test for independent samples; and paired t test and McNemar’s test for paired samples) were used to compare continuous and nominal data between the CMT cohort and the comparison group before and after matching. Participants with missing values were excluded from the analysis.

## Results

### Comparisons between CMT and Vancouver Provincial Court

We first examined differences between CMT participants and members of the available comparison group from the Provincial court (n = 4,377). Within the DCC cohort, a total of 279 individuals were triaged to CMT and exited the program prior to March 31 2012. Results indicate that members of the CMT cohort were older (p = 0.015), more likely to be female (p = 0.011), more likely to be Aboriginal (p<0.001), and less well educated than those seen in the Vancouver Provincial Court (p<0.001; see Table 1).

**Table 1:** Comparison of Socio-demographic characteristics between Case Management Team and Vancouver Provincial Court offenders.

Variables	Specifier	Case Management Team n = 279	Provincial Court Participants n = 4377	P value
Age at enrolment in years	Mean (SD)	37.8 (10.2)	36.1 (11.2)	**0.015**
Gender	Male	221 (79)	3715 (85)	**0.011**
	Female	58 (21)	662 (15)	
Ethnicity	Caucasian	179 (66)	2295 (55)	**<0.001**
	Aboriginal	60 (22)	605 (15)	
	Other	33 (12)	1248 (30)	
Education level	Grade 9 or less	49 (19)	477 (12)	**<0.001**
	Grade 10/11	95 (37)	1186 (31)	
	Grade 12	89 (34)	1430 (37)	
	Vocational/University	27 (10)	749 (20)	
Date of Enrolment	Mean (Min, Max)	August 2009 (May 2008, November 2011)	July 2009 (April 2008, March 2011)	

Additional differences were observed when we compared CMT and Vancouver Provincial Court offenders on several corrections-related characteristics. Members of the CMT cohort had committed significantly more offences overall (p<0.001), and when examined over a ten year period, significantly more offences of various sub-types, including property (p<0.001), breach (p<0.001), weapons (p<0.038) and violence-related offences (p<0.001), and sentences that involved jail (p<0.001; see Table 2).

**Table 2:** Comparison of Corrections related characteristics between Case Management Team and Vancouver Provincial Court offenders.

Variables	Period of Time	Case Management Team n = 279	Provincial Court Participants n = 4377	P value
		Mean (SD)	Mean (SD)	
Number of offences (any)	Last 10 years	16.9 (17.0)	6.4 (9.2)	**<0.001**
	Last 5 years	10.0 (9.2)	4.2 (5.3)	**<0.001**
	Last 2 years	5.3 (4.8)	2.5 (2.7)	**<0.001**
	Last year	3.5 (2.8)	1.6 (2.0)	**<0.001**
Number of breach offences	Last 10 years	4.0 (5.3)	1.3 (2.8)	**<0.001**
	Last 5 years	2.5 (3.5)	0.9 (1.8)	**<0.001**
	Last 2 years	1.4 (2.2)	0.5 (1.1)	**<0.001**
	Last year	0.9 (1.4)	0.3 (0.8)	**<0.001**
Number of property offences	Last 10 years	9.4 (12.5)	2.8 (5.9)	**<0.001**
	Last 5 years	5.4 (6.4)	1.7 (3.5)	**<0.001**
	Last 2 years	2.9 (3.1)	0.9 (1.8)	**<0.001**
	Last year	2.0 (2.0)	0.6 (1.3)	**<0.001**
Number of weapon offences	Last 10 years	0.2 (0.6)	0.1 (0.7)	**0.0381**
	Last 5 years	0.1 (0.4)	0.1 (0.4)	0.159
	Last 2 years	0.1 (0.4)	0.1 (0.3)	0.361
	Last year	0.1 (0.3)	<0.1 (0.2)	0.173
Number of violent offences	Last 10 years	1.6 (2.3)	1.0 (2.0)	**<0.001**
	Last 5 years	1.0 (1.4)	0.7 (1.4)	**0.004**
	Last 2 years	0.6 (0.9)	0.5 (1.1)	0.451
	Last year	0.4 (0.7)	0.3 (0.9)	0.451
Number of jail sentences	Last 10 years	11.2 (13.4)	3.8 (7.5)	**<0.001**
	Last 5 years	6.9 (8.1)	2.5 (4.7)	**<0.001**
	Last 2 years	3.4 (4.6)	1.5 (2.8)	**<0.001**
	Last year	1.8 (2.5)	1.1 (2.2)	**<0.001**

We next compared CMT and Vancouver Provincial Court participants on a number of health and social assistance domains, finding significantly higher levels of service use among members of the CMT cohort in each instance and over every time period examined (between one and ten years). Individuals triaged to CMT had higher numbers of visits to medical doctors, higher payments associated with physician care, a higher number of hospital admissions, greater numbers of days spent in hospital, and were paid higher amounts of social assistance (p<0.001 for all; see Table 3).

**Table 3:** Comparison of health Care and social service utilization between Case Management Team and Vancouver Provincial Court offenders.

Variables	Period of Time	Case Management Team n = 279	Provincial Court Participants n = 4377	P value
		Mean (SD)	Mean (SD)	
Medical Services Plan payments ($CAD)	Last 10 years	9141 (10208)	5001 (6826)	**<0.001**
	Last 5 years	5614 (6663)	2862 (4184)	**<0.001**
	Last 2 years	2883 (4218)	1324 (2253)	**<0.001**
	Last year	1471 (2088)	709 (1387)	**<0.001**
# of Medical Services Plan services	Last 10 years	295 (359)	171 (279)	**<0.001**
	Last 5 years	179 (215)	102 (167)	**<0.001**
	Last 2 years	81 (99)	46 (75)	**<0.001**
	Last year	40 (50)	24 (42)	**<0.001**
# of hospital admissions (acute)	Last 10 years	3.9 (6.2)	1.2 (2.8)	**<0.001**
	Last 5 years	2.8 (4.7)	0.8 (2.0)	**<0.001**
	Last 2 years	1.5 (3.0)	0.4 (1.3)	**<0.001**
	Last year	0.8 (1.6)	0.2 (0.8)	**<0.001**
Number of hospital days	Last 10 years	30.4 (61.2)	9.0 (30.5)	**<0.001**
	Last 5 years	23.0 (50.0)	6.0 (21.5)	**<0.001**
	Last 2 years	12.4 (31.5)	3.0 (13.3)	**<0.001**
	Last year	6.1 (15.8)	1.7 (9.2)	**<0.001**
Social assistance payments ($CAD)	Last 10 years	41238 (38170)	22907 (34601)	**<0.001**
	Last 5 years	23997 (20563)	11583 (18786)	**<0.001**
	Last 2 years	11421 (8936)	5594 (8463)	**<0.001**
	Last year	5892 (4454)	3040 (4468)	**<0.001**

### Matching and Recidivism Outcomes

A Matched Comparison Group (MCG) was created using a wide range of sociodemographic, criminal justice, health and social assistance variables. The matching process was implemented to minimize differences between CMT and Vancouver Provincial Court participants presented above. Twenty-three matching variables were selected with an emphasis on factors known to have an influence on offence-related risk, including: age; ethnicity; education level; overall prior offending; violent offending; offences involving weapons; time in jail; mental disorders including substance use disorders; and income assistance. Physician services are provided on a fee for service basis and paid under the publicly administered Medical Services Plan (MSP). MSP services include diagnostic information as well as the amount paid. Details collected under MSP are included in Tables 3 and 4.

**Table 4:** Comparison of socio-demographics, justice and community service use between Case Management Team and Matched Comparison Group.

Variables	Case Management Teamn = 249	Matched Comparison Groupn = 249	P value^1^
	Mean (SD) or N (%)	Mean (SD) or N (%)	
Age at enrolment in years	37.7 (10.1)	36.7 (9.2)	0.212
Gender (male vs. female)	199 (80)	200 (80)	0.912
Caucasian ethnicity (yes vs. no)	163 (65)	163 (65)	1.00
Aboriginal ethnicity (yes vs. no)	56 (23)	62 (25)	0.522
Other ethnicity (yes vs. no)	30 (12)	24 (10)	0.387
Gd. 9 or less education (yes vs. no)	45 (18)	40 (16)	0.535
Gd. 10 or 11 education (yes vs. no)	93 (37)	93 (37)	1.00
Gd. 12 education (yes vs. no)	85 (34)	96 (39)	0.301
University/vocational education (yes vs. no)	26 (11)	20 (8)	0.343
# of any offences (last 10 years)	16.1 (15.4)	16.9 (16.2)	0.483
# of property offences (last 10 years)	8.7 (11.0)	9.0 (11.9)	0.756
# of breach offences (last 10 years)	3.8 (5.1)	4.1 (5.0)	0.521
# of weapon offences (last 10 years)	0.2 (0.7)	0.3 (1.2)	0.459
# of violent offences (last 10 years)	1.6 (2.3)	1.7 (2.8)	0.642
# of jail sentences (last 10 years)	10.7 (12.6)	11.4 (13.2)	0.508
# of Medical Services Plan (MSP) services (last 5 years)	178.3 (219.2)	185.8 (229.5)	0.694
MSP payments (last 5 years)	5373 (6430)	5009 (5417)	0.440
# of MSP encounters for Non-Substance related Mental Disorder (last 5 years)	19.2 (47.6)	22.5 (59.9)	0.478
# of MSP encounters for Substance related Mental Disorder (last 5 years)	33.6 (73.5)	29.7 (76.1)	0.561
Acute hospital admission (last 5 years)	2.6 (4.3)	2.1 (3.8)	0.186
# of hospital days (last 5 years)	19.9 (42.3)	17.7 (40.8)	0.507
# of social assistance payments (last 5 years)	35.4 (21.6)	35.7 (22.4)	0.832
Social assistance payments (last 5 years)	24814 (21010)	26011 (20970)	0.479

1 Paired t test was used to compare continuous variables and McNemar test was used to compare categorical variables.

A total of 249 individuals were matched simultaneously on all variables (see Table 4). Modest attrition from the total CMT sample (n = 279) was due to missing data for any one of the variables included in the simultaneous matching procedure. We compared the frequency of custody sentences between the two groups in order to test for potential non-equivalence of time at risk and found no significant difference (CMT vs. MCG: 0.95 vs. 0.93, p = 0.889). The mean duration of involvement with the CMT was 368 days (SD: 275 days).

We examined the number of offences in the pre-period compared with the number of offences in the post period. Each recorded conviction was counted as one offence, regardless of the number of charges. A difference score was calculated, with a positive value representing a reduction in offending. Members of the CMT cohort demonstrated a significantly greater reduction in offending compared to the MCG (see Table 5). Those in CMT had a mean reduction of 2.27 offences per person versus 1.34 per person in the MCG (p<0.001). The observed overall reduction in offending was sustained in large part by a significant reduction in property offences (1.35 versus 0.55; p<0.001).

**Table 5:** Comparison of outcome measure (reduction in offences) between Case Management Team and Matched Comparison Group.^1^

Variables	Case Management Team n = 249	Matched Comparison Group n = 249	P value^2^
	Mean (SD)	Mean (SD)	
Number of any offences (Reduction per person per year)	2.27 (2.90)	1.34 (2.54)	**<0.001**
Number of property offences (Reduction per person per year)	1.35 (1.94)	0.55 (1.44)	**<0.001**
Number of breach offences (Reduction per person per year)	0.50 (1.35)	0.29 (1.47)	0.110
Number of violent offences (Reduction per person per year)	0.22 (0.88)	0.21 (1.08)	0.929

1 Reduction in offence was calculated from the difference of offence between 1-year pre and 1 year post period.

2 Paired t test was used to compare continuous outcome variables.

## Discussion

Individuals assigned to the DCC’s Case Management Teams (CMT) exhibited significantly greater reductions in reoffending compared to a matched group of Provincial offenders that received traditional justice responses. Reductions in offending were primarily associated with property crimes and breach offences, both of which have multiple impacts on perceptions of neighbourhood safety as well as justice system resources [20]. In each of these two offence categories, the CMT cohort exhibited reductions approximately twice as large as those observed in the comparison group. These findings suggest that with appropriate supports, this type of response can produce significantly greater reductions in crime than traditional responses to offenders with complex needs.

Our study represents the first empirical examination of a community court on recidivism, and our findings await confirmation from research in other jurisdictions. Nevertheless, our results are consistent with those of studies addressing other, better-established forms of problem solving courts, such as Drug Treatment Courts [21] and Mental Health Courts [22], [23]. In addition, research indicates that the impact of problem solving courts on recidivism is greater when they include effective triage practices and match offenders to interventions following the principles of Risk-Need-Responsivity [24], [25]. The complexity of need in the CMT cohort was illustrated when we compared the cohort to offenders in the nearby Vancouver Provincial Court. Those triaged to CMT were significantly higher users of all public services examined, including community medicine, hospitals, financial assistance and social support, and justice services. Moreover, these differences were evident over periods of time extending retrospectively for ten years. Apart from the diversity and longstanding duration of need within the CMT cohort, observed differences between CMT and other DCC clients included variables specifically associated with criminogenic risk, such as significantly higher numbers of violent offences, jail sentences, and offences involving weapons [26].

We used the propensity score matching method [27] to create a comparison group that received traditional court processing in the same location and within the same period of time as the CMT cohort. Matching included an array of variables representing sociodemographic characteristics, health service use, social welfare receipt, and different forms of justice system involvement. It is impossible to control for all potential differences between groups using non-experimental techniques. However, PSM has been widely used in circumstances where randomization is either impractical or otherwise precluded. The present study includes one of the most comprehensive matching procedures used in the context of research on problem solving courts.

Considerably more research is needed on problem solving courts generally, and on community courts in particular [8]. It is unknown whether community courts would be similarly effective in settings that vary based on size or other contextual features. It is also unknown what adaptations may be needed in order to meet the needs of subgroups of offenders, such as those supported by the CMT. Furthermore, it is important to examine whether investments made in particular programs via community courts are offset by any savings associated with reductions in service or changes in the type of service use or by other financial benefits to society. A growing body of research indicates that public expenditures can be avoided by justice programs that divert offenders from patterns of costly and cyclical service use [28], [29]. Over the ten years before their index offence, members of the CMT cohort incurred two and a half times the number of offences, double the amount paid in medical visits, three times as many days in hospital, and double the financial assistance of offenders in the Vancouver Provincial Court. These findings strongly suggest the opportunity for cost avoidance. However, whether cost avoidance is realized by CMT, and how this compares to the costs of intervening is unknown at this point.

The present study focused on offenders triaged to CMT, primarily because this subgroup committed the highest number of offences per capita, presented the most complex criminogenic needs, and therefore represented the greatest opportunity for the DCC to fulfill its goal of reducing crime, promoting positive offender outcomes, and improving community safety. The CMT cohort itself was small enough in number (less than 300) that it was possible to identify a matched group from within the population seen in the neighbouring Vancouver Provincial Court. Having identified encouraging results with this sub-group of frequent offenders, it is important to examine whether the DCC is similarly effective using less intensive court resources with lower risk individuals. The Vancouver DCC hears approximately 4,500 court cases involving over 2,500 unique individuals per year, meaning that the use of propensity score matching may require an eligible sample from multiple jurisdictions in order to have sufficient numbers to identify cases that match the characteristics of the large numbers of offenders seen in the DCC.

The resources of the CMT were designed following extensive planning within government, the judiciary, and other agencies, and reflect the diverse needs of the client population. The CMT provides a diverse inter-professional team and a variety of community resources. Although effective, the CMT is a “black box” and we are unable to evaluate whether particular elements of the team are responsible for the observed outcomes, or if specific services are more effective for sub-groups of offenders. Qualitative interviews with offenders, CMT members, and community key informants may help distinguish the elements of CMT involvement that are most effective, and perhaps identify areas that would benefit from further growth and development.

The fact that participants were not randomly assigned to CMT or Vancouver Provincial Court means that we cannot assume complete equivalence between the two groups. Nevertheless, our design included a large range of matching variables representing both static and dynamic risk factors for recidivism. We examined re-offence rates over a one year period, including all Provincial sentences. A longer period of follow up may be important, to confirm the stability of changes in recidivism, and as a basis for research on cost avoidance and cost effectiveness. We did not control for time spent in hospital or other institutional settings during the follow up period. However, we controlled for multiple health and social factors in creating the comparison group and we are aware of no reason that the two cohorts would differ in their respective time at risk (i.e., the number of days available to commit offences) in the follow up period.

The present study is the first empirical research examining the impact of a community court on recidivism. Our findings indicate that the DCC produced significantly greater reductions in offending compared to traditional adjudication among a sub-group of offenders with extensive criminal, healthcare, and social service use histories. In addition, the individuals included in our analysis were differentiated from the general offender population on the basis of several sociodemographic factors, including a higher prevalence of females, Aboriginal peoples, and people with lower educational achievement. By coordinating inter-agency and community resources, the CMT illustrates that both health and public safety improvements emerge from interacting social determinants [30]. These results add to the body of research that supports the effectiveness of problem solving courts, and now await replication in other jurisdictions to confirm the robustness of the community court model.
